# *N*-{2-[4-(2-Hy­droxy­eth­yl)piperazin-1-yl]eth­yl}phthalimide

**DOI:** 10.1107/S160053681105327X

**Published:** 2011-12-21

**Authors:** Ying Shao, Dong An, Mi Zhou, Li Liu, Xiao-Qiang Sun

**Affiliations:** a Key Laboratory of Fine Petrohemical Engineering, Changzhou University, Changzhou 213164, Jiangsu, People’s Republic of China; b Analytical Center, Changzhou University, Changzhou 213164, People’s Republic of China

## Abstract

In the title compound, C_16_H_21_N_3_O_3_, the piperazine ring adopts a chair conformation, with its N—C bonds in pseudo-equatorial orientations. In the crystal, mol­ecules are linked by O—H⋯N hydrogen bonds, generating *C*(5) chains propagating in [101]. Weak aromatic π–π stacking inter­actions also occur [centroid–centroid separation = 3.899 (1) Å].

## Related literature

For general background to piperazine derivatives, see: Tian *et al.* (2011 ▶). For the preparation, see: Ghosh *et al.* (2010 ▶).
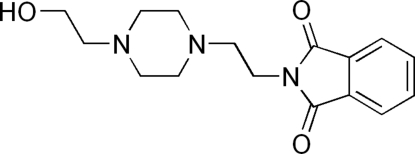


## Experimental

### 

#### Crystal data

C_16_H_21_N_3_O_3_
*M* *_r_* = 303.36
Monoclinic, 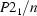

*a* = 5.8109 (6) Å
*b* = 37.012 (4) Å
*c* = 7.3537 (8) Å
β = 95.634 (2)°
*V* = 1573.9 (3) Å^3^
*Z* = 4
Mo *K*α radiation
μ = 0.09 mm^−1^
*T* = 296 K
0.25 × 0.22 × 0.20 mm

#### Data collection

Bruker APEXII CCD diffractometer
Absorption correction: multi-scan (*SADABS*; Bruker, 2000 ▶) *T*_min_ = 0.978, *T*_max_ = 0.982
8562 measured reflections
2775 independent reflections
2537 reflections with *I* > 2σ(*I*)
*R*_int_ = 0.025

#### Refinement

*R*[*F* ^2^ > 2σ(*F* ^2^)] = 0.057
*wR*(*F* ^2^) = 0.161
*S* = 1.00
2775 reflections
201 parameters
H-atom parameters constrained
Δρ_max_ = 0.53 e Å^−3^
Δρ_min_ = −0.38 e Å^−3^

### 

Data collection: *APEX2* (Bruker, 2000 ▶); cell refinement: *SAINT* (Bruker, 2000 ▶); data reduction: *SAINT*; program(s) used to solve structure: *SHELXS97* (Sheldrick, 2008 ▶); program(s) used to refine structure: *SHELXL97* (Sheldrick, 2008 ▶); molecular graphics: *SHELXTL* (Sheldrick, 2008 ▶); software used to prepare material for publication: *SHELXTL*.

## Supplementary Material

Crystal structure: contains datablock(s) I, global. DOI: 10.1107/S160053681105327X/hb6564sup1.cif

Structure factors: contains datablock(s) I. DOI: 10.1107/S160053681105327X/hb6564Isup2.hkl

Supplementary material file. DOI: 10.1107/S160053681105327X/hb6564Isup3.cdx

Supplementary material file. DOI: 10.1107/S160053681105327X/hb6564Isup4.cml

Additional supplementary materials:  crystallographic information; 3D view; checkCIF report

## Figures and Tables

**Table 1:** Hydrogen-bond geometry (Å, °)

*D*—H⋯*A*	*D*—H	H⋯*A*	*D*⋯*A*	*D*—H⋯*A*
O3—H3*A*⋯N3^i^	0.82	2.00	2.811 (3)	171

Symmetry code: (i) 
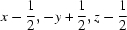
.

**Fig. 1.**
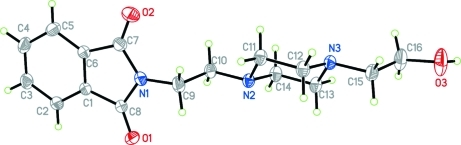
View of the title compound, showing 50% probability ellipsoids.

**Fig. 2.**
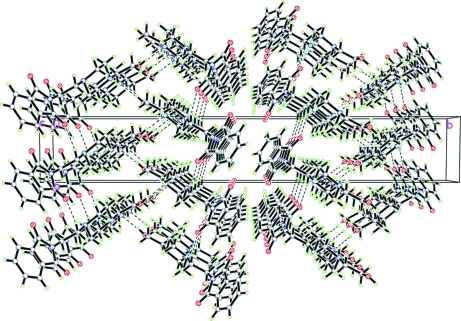
Perspective view of the title compound along c direction. Labels of atoms have been omitted for clarity.

## supplementary crystallographic information

### Comment

Piperazine derivatives in pharmaceutical and chemical industries have a wide range of applications (Tian, *et al.*, 2011). The title compound, which is an intermediate for our designed drug, was synthesized from 2-(2-bromoethyl)phthalimide and 2-(piperazin-1-yl)ethanol in the presence of K_2_CO_3_ as acid-acceptor (Ghosh, *et al.*, 2010). In the molecule of the title compound (Fig. 1), the phthalimide fragment is planar, with r.m.s. deviation of 0.02 Å. The six-membered piperazine ring adopts the chair conformation. In the crystal, the crystal packing is stabilized by O—H···N hydrogen bonding interactions and π–π interactions involving the benzene ring (Fig. 2). For the O—H···N the hydrogen bonding interactions, the relevant distances and angles are: O3···H3*A*= 0.821 Å, H3*A*···N3 =1.997 Å, O3···N3^[i]^= 2.811 (3) Å, and O3—H3*A*···N3^[i]^= 171.3°. And for the π ···π interactions, the relevant distances are: *Cg*···*Cg*^[ii]^ = 3.899 (1)Å [symmetry code: (i) *x* - 1/2, 1/2 - *y*, -1/2 + *z*; (ii) 2 - *x*, -*y*, 2 - *z*; *Cg* is the centroid of the C1–C2–C3–C4–C5–C6 ring].

### Experimental

A suspension of 2-(2-bromoethyl)phthalimide (0.63 g, 2.5 mmol), 2-(piperazin-1-yl)ethanol (0.36 g, 2.8 mmol) and K_2_CO_3_ (0.90 g, 6.5 mmol) in 15 ml acetonitrile was stirred at room temperature for 0.5 h, and then heated to reflux for 10 h. After cooling and filtration, the filter residue was washed with CH_3_CN. And the filtrate and washing were combined prior to removing the solvent under vacuum. A white powder (0.55 g, 1.8 mmol) was obtained after recrystallization from ethyl acetate/ petroleum ether. Colourless blocks were obtained by slow evaporation of a CH_3_OH solution.

### Refinement

All the H atoms were placed in geometrically idealized positions and constrained to ride on their parent atoms, with C—H distances of 0.93–0.97 Å, and with *U*_iso_(H) = 1.2*U*_eq_(C).

### Crystal data

**Table:**

C_16_H_21_N_3_O_3_	*F*(000) = 648
*M**_r_* = 303.36	*D*_x_ = 1.280 Mg m^−^^3^
Monoclinic, *P*2_1_/*n*	Mo *K*α radiation, λ = 0.71073 Å
Hall symbol: -P 2yn	Cell parameters from 5733 reflections
*a* = 5.8109 (6) Å	θ = 2.8–29.9°
*b* = 37.012 (4) Å	µ = 0.09 mm^−^^1^
*c* = 7.3537 (8) Å	*T* = 296 K
β = 95.634 (2)°	Block, colorless
*V* = 1573.9 (3) Å^3^	0.25 × 0.22 × 0.20 mm
*Z* = 4	

### Data collection

**Table:**

Bruker APEXII CCD diffractometer	2775 independent reflections
Radiation source: fine-focus sealed tube	2537 reflections with *I* > 2σ(*I*)
graphite	*R*_int_ = 0.025
φ and ω scans	θ_max_ = 25.1°, θ_min_ = 2.2°
Absorption correction: multi-scan (*SADABS*; Bruker, 2000)	*h* = −6→6
*T*_min_ = 0.978, *T*_max_ = 0.982	*k* = −44→43
8562 measured reflections	*l* = −8→6

### Refinement

**Table:**

Refinement on *F*^2^	Secondary atom site location: difference Fourier map
Least-squares matrix: full	Hydrogen site location: inferred from neighbouring sites
*R*[*F*^2^ > 2σ(*F*^2^)] = 0.057	H-atom parameters constrained
*wR*(*F*^2^) = 0.161	*w* = 1/[σ^2^(*F*_o_^2^) + (0.081*P*)^2^ + 1.190*P*] where *P* = (*F*_o_^2^ + 2*F*_c_^2^)/3
*S* = 1.00	(Δ/σ)_max_ < 0.001
2775 reflections	Δρ_max_ = 0.53 e Å^−^^3^
201 parameters	Δρ_min_ = −0.38 e Å^−^^3^
0 restraints	Extinction correction: *SHELXL97* (Sheldrick, 2008), Fc^*^=kFc[1+0.001xFc^2^λ^3^/sin(2θ)]^-1/4^
Primary atom site location: structure-invariant direct methods	Extinction coefficient: 0.024 (4)

### Special details

**Table:**

Geometry. All e.s.d.'s (except the e.s.d. in the dihedral angle between two l.s. planes) are estimated using the full covariance matrix. The cell e.s.d.'s are taken into account individually in the estimation of e.s.d.'s in distances, angles and torsion angles; correlations between e.s.d.'s in cell parameters are only used when they are defined by crystal symmetry. An approximate (isotropic) treatment of cell e.s.d.'s is used for estimating e.s.d.'s involving l.s. planes.
Refinement. Refinement of *F*^2^ against ALL reflections. The weighted *R*-factor *wR* and goodness of fit *S* are based on *F*^2^, conventional *R*-factors *R* are based on *F*, with *F* set to zero for negative *F*^2^. The threshold expression of *F*^2^ > σ(*F*^2^) is used only for calculating *R*-factors(gt) *etc*. and is not relevant to the choice of reflections for refinement. *R*-factors based on *F*^2^ are statistically about twice as large as those based on *F*, and *R*- factors based on ALL data will be even larger.

### Fractional atomic coordinates and isotropic or equivalent isotropic displacement parameters (Å^2^)

**Table:**

	*x*	*y*	*z*	*U*_iso_*/*U*_eq_	
C1	0.8296 (4)	0.04878 (5)	0.9736 (3)	0.0374 (5)	
C2	0.7865 (4)	0.02500 (6)	1.1105 (3)	0.0471 (6)	
H2	0.6530	0.0110	1.1024	0.057*	
C3	0.9495 (5)	0.02280 (7)	1.2607 (3)	0.0522 (6)	
H3	0.9260	0.0067	1.3543	0.063*	
C4	1.1458 (5)	0.04391 (7)	1.2744 (3)	0.0538 (6)	
H4	1.2518	0.0419	1.3773	0.065*	
C5	1.1882 (4)	0.06811 (7)	1.1375 (3)	0.0496 (6)	
H5	1.3199	0.0825	1.1466	0.060*	
C6	1.0265 (4)	0.06991 (6)	0.9870 (3)	0.0389 (5)	
C7	1.0250 (4)	0.09147 (6)	0.8160 (3)	0.0429 (5)	
C8	0.6974 (4)	0.05594 (6)	0.7936 (3)	0.0414 (5)	
C9	0.7626 (5)	0.09520 (6)	0.5245 (3)	0.0473 (6)	
H9A	0.6659	0.0776	0.4558	0.057*	
H9B	0.9024	0.0982	0.4640	0.057*	
C10	0.6356 (4)	0.13096 (6)	0.5228 (3)	0.0398 (5)	
H10A	0.5043	0.1288	0.5940	0.048*	
H10B	0.7380	0.1494	0.5789	0.048*	
C11	0.7464 (4)	0.15193 (6)	0.2321 (3)	0.0412 (5)	
H11A	0.8526	0.1318	0.2290	0.049*	
H11B	0.8300	0.1721	0.2916	0.049*	
C12	0.6585 (4)	0.16244 (6)	0.0396 (3)	0.0432 (5)	
H12A	0.7881	0.1687	−0.0280	0.052*	
H12B	0.5785	0.1421	−0.0210	0.052*	
C13	0.3083 (4)	0.18340 (7)	0.1460 (3)	0.0472 (6)	
H13A	0.2223	0.1635	0.0865	0.057*	
H13B	0.2044	0.2038	0.1501	0.057*	
C14	0.3970 (4)	0.17252 (6)	0.3390 (3)	0.0439 (5)	
H14A	0.4768	0.1928	0.4006	0.053*	
H14B	0.2676	0.1661	0.4064	0.053*	
C15	0.4241 (5)	0.20357 (6)	−0.1491 (3)	0.0519 (6)	
H15A	0.3210	0.1850	−0.2032	0.062*	
H15B	0.5576	0.2045	−0.2184	0.062*	
C16	0.3036 (6)	0.23880 (8)	−0.1657 (4)	0.0696 (8)	
H16A	0.1483	0.2367	−0.1290	0.083*	
H16B	0.3870	0.2568	−0.0892	0.083*	
N1	0.8237 (3)	0.08156 (5)	0.7086 (2)	0.0419 (5)	
N2	0.5552 (3)	0.14180 (5)	0.3366 (2)	0.0360 (4)	
N3	0.5012 (3)	0.19317 (5)	0.0404 (2)	0.0403 (5)	
O1	0.5199 (3)	0.04208 (5)	0.7274 (2)	0.0618 (5)	
O2	1.1636 (3)	0.11320 (5)	0.7720 (3)	0.0648 (6)	
O3	0.2965 (5)	0.24873 (7)	−0.3547 (3)	0.0921 (8)	
H3A	0.2018	0.2650	−0.3766	0.138*	

### Atomic displacement parameters (Å^2^)

**Table:**

	*U*^11^	*U*^22^	*U*^33^	*U*^12^	*U*^13^	*U*^23^
C1	0.0419 (11)	0.0314 (10)	0.0381 (11)	0.0005 (8)	−0.0002 (9)	0.0024 (8)
C2	0.0529 (14)	0.0431 (12)	0.0443 (12)	−0.0071 (10)	−0.0002 (10)	0.0086 (10)
C3	0.0676 (16)	0.0493 (13)	0.0383 (12)	0.0031 (12)	−0.0014 (11)	0.0086 (10)
C4	0.0582 (15)	0.0584 (15)	0.0416 (13)	0.0088 (12)	−0.0115 (11)	0.0004 (11)
C5	0.0417 (12)	0.0526 (14)	0.0524 (14)	−0.0020 (10)	−0.0059 (10)	−0.0047 (11)
C6	0.0398 (11)	0.0342 (10)	0.0420 (12)	0.0016 (8)	0.0011 (9)	0.0001 (9)
C7	0.0434 (12)	0.0382 (11)	0.0473 (12)	−0.0015 (9)	0.0054 (10)	0.0020 (9)
C8	0.0452 (12)	0.0343 (11)	0.0432 (12)	−0.0016 (9)	−0.0034 (9)	0.0041 (9)
C9	0.0655 (15)	0.0407 (12)	0.0354 (11)	0.0044 (10)	0.0033 (10)	0.0052 (9)
C10	0.0457 (12)	0.0431 (12)	0.0310 (10)	0.0030 (9)	0.0059 (9)	0.0049 (8)
C11	0.0380 (11)	0.0497 (12)	0.0364 (11)	0.0053 (9)	0.0067 (9)	0.0062 (9)
C12	0.0481 (12)	0.0487 (13)	0.0335 (11)	0.0026 (10)	0.0076 (9)	0.0035 (9)
C13	0.0440 (12)	0.0513 (13)	0.0448 (13)	0.0093 (10)	−0.0025 (10)	0.0073 (10)
C14	0.0417 (12)	0.0531 (13)	0.0375 (12)	0.0081 (10)	0.0067 (9)	0.0072 (10)
C15	0.0728 (17)	0.0457 (13)	0.0350 (12)	0.0036 (12)	−0.0063 (11)	0.0025 (10)
C16	0.098 (2)	0.0624 (17)	0.0448 (14)	0.0265 (16)	−0.0084 (14)	0.0069 (12)
N1	0.0491 (11)	0.0367 (9)	0.0391 (10)	−0.0018 (8)	0.0011 (8)	0.0075 (8)
N2	0.0373 (9)	0.0403 (9)	0.0307 (9)	0.0014 (7)	0.0046 (7)	0.0047 (7)
N3	0.0502 (11)	0.0404 (10)	0.0291 (9)	−0.0017 (8)	−0.0018 (7)	0.0031 (7)
O1	0.0588 (11)	0.0610 (11)	0.0604 (11)	−0.0196 (9)	−0.0195 (9)	0.0150 (9)
O2	0.0597 (11)	0.0635 (11)	0.0720 (13)	−0.0211 (9)	0.0098 (9)	0.0157 (9)
O3	0.136 (2)	0.0818 (15)	0.0558 (12)	0.0490 (15)	−0.0048 (13)	0.0216 (11)

### Geometric parameters (Å, °)

**Table:**

C1—C2	1.379 (3)	C11—N2	1.460 (3)
C1—C6	1.381 (3)	C11—C12	1.508 (3)
C1—C8	1.487 (3)	C11—H11A	0.9700
C2—C3	1.385 (3)	C11—H11B	0.9700
C2—H2	0.9300	C12—N3	1.460 (3)
C3—C4	1.378 (4)	C12—H12A	0.9700
C3—H3	0.9300	C12—H12B	0.9700
C4—C5	1.387 (4)	C13—N3	1.470 (3)
C4—H4	0.9300	C13—C14	1.516 (3)
C5—C6	1.381 (3)	C13—H13A	0.9700
C5—H5	0.9300	C13—H13B	0.9700
C6—C7	1.489 (3)	C14—N2	1.463 (3)
C7—O2	1.204 (3)	C14—H14A	0.9700
C7—N1	1.395 (3)	C14—H14B	0.9700
C8—O1	1.210 (3)	C15—N3	1.472 (3)
C8—N1	1.385 (3)	C15—C16	1.479 (4)
C9—N1	1.456 (3)	C15—H15A	0.9700
C9—C10	1.515 (3)	C15—H15B	0.9700
C9—H9A	0.9700	C16—O3	1.435 (3)
C9—H9B	0.9700	C16—H16A	0.9700
C10—N2	1.459 (3)	C16—H16B	0.9700
C10—H10A	0.9700	O3—H3A	0.8200
C10—H10B	0.9700		
			
C2—C1—C6	121.2 (2)	H11A—C11—H11B	108.1
C2—C1—C8	130.4 (2)	N3—C12—C11	110.60 (17)
C6—C1—C8	108.36 (18)	N3—C12—H12A	109.5
C1—C2—C3	117.4 (2)	C11—C12—H12A	109.5
C1—C2—H2	121.3	N3—C12—H12B	109.5
C3—C2—H2	121.3	C11—C12—H12B	109.5
C4—C3—C2	121.5 (2)	H12A—C12—H12B	108.1
C4—C3—H3	119.3	N3—C13—C14	110.70 (18)
C2—C3—H3	119.3	N3—C13—H13A	109.5
C3—C4—C5	121.1 (2)	C14—C13—H13A	109.5
C3—C4—H4	119.4	N3—C13—H13B	109.5
C5—C4—H4	119.4	C14—C13—H13B	109.5
C6—C5—C4	117.2 (2)	H13A—C13—H13B	108.1
C6—C5—H5	121.4	N2—C14—C13	110.55 (18)
C4—C5—H5	121.4	N2—C14—H14A	109.5
C5—C6—C1	121.6 (2)	C13—C14—H14A	109.5
C5—C6—C7	130.5 (2)	N2—C14—H14B	109.5
C1—C6—C7	107.87 (18)	C13—C14—H14B	109.5
O2—C7—N1	124.7 (2)	H14A—C14—H14B	108.1
O2—C7—C6	129.5 (2)	N3—C15—C16	114.0 (2)
N1—C7—C6	105.80 (18)	N3—C15—H15A	108.8
O1—C8—N1	125.2 (2)	C16—C15—H15A	108.8
O1—C8—C1	128.9 (2)	N3—C15—H15B	108.8
N1—C8—C1	105.84 (17)	C16—C15—H15B	108.8
N1—C9—C10	112.62 (18)	H15A—C15—H15B	107.7
N1—C9—H9A	109.1	O3—C16—C15	105.9 (2)
C10—C9—H9A	109.1	O3—C16—H16A	110.6
N1—C9—H9B	109.1	C15—C16—H16A	110.6
C10—C9—H9B	109.1	O3—C16—H16B	110.6
H9A—C9—H9B	107.8	C15—C16—H16B	110.6
N2—C10—C9	111.02 (17)	H16A—C16—H16B	108.7
N2—C10—H10A	109.4	C8—N1—C7	112.13 (18)
C9—C10—H10A	109.4	C8—N1—C9	124.43 (19)
N2—C10—H10B	109.4	C7—N1—C9	123.34 (19)
C9—C10—H10B	109.4	C11—N2—C10	111.95 (16)
H10A—C10—H10B	108.0	C11—N2—C14	108.56 (17)
N2—C11—C12	110.79 (18)	C10—N2—C14	110.22 (16)
N2—C11—H11A	109.5	C12—N3—C13	108.70 (17)
C12—C11—H11A	109.5	C12—N3—C15	109.44 (17)
N2—C11—H11B	109.5	C13—N3—C15	112.78 (18)
C12—C11—H11B	109.5	C16—O3—H3A	109.5
			
C6—C1—C2—C3	−0.8 (3)	O1—C8—N1—C7	−177.6 (2)
C8—C1—C2—C3	176.4 (2)	C1—C8—N1—C7	0.3 (2)
C1—C2—C3—C4	1.0 (4)	O1—C8—N1—C9	−1.1 (4)
C2—C3—C4—C5	−0.3 (4)	C1—C8—N1—C9	176.87 (19)
C3—C4—C5—C6	−0.4 (4)	O2—C7—N1—C8	179.9 (2)
C4—C5—C6—C1	0.5 (3)	C6—C7—N1—C8	−0.1 (2)
C4—C5—C6—C7	−177.1 (2)	O2—C7—N1—C9	3.2 (4)
C2—C1—C6—C5	0.1 (3)	C6—C7—N1—C9	−176.69 (19)
C8—C1—C6—C5	−177.7 (2)	C10—C9—N1—C8	97.9 (3)
C2—C1—C6—C7	178.2 (2)	C10—C9—N1—C7	−85.9 (3)
C8—C1—C6—C7	0.4 (2)	C12—C11—N2—C10	179.14 (17)
C5—C6—C7—O2	−2.3 (4)	C12—C11—N2—C14	−59.0 (2)
C1—C6—C7—O2	179.9 (2)	C9—C10—N2—C11	−69.7 (2)
C5—C6—C7—N1	177.6 (2)	C9—C10—N2—C14	169.38 (19)
C1—C6—C7—N1	−0.2 (2)	C13—C14—N2—C11	58.3 (2)
C2—C1—C8—O1	−0.1 (4)	C13—C14—N2—C10	−178.72 (18)
C6—C1—C8—O1	177.4 (2)	C11—C12—N3—C13	−58.2 (2)
C2—C1—C8—N1	−178.0 (2)	C11—C12—N3—C15	178.21 (19)
C6—C1—C8—N1	−0.4 (2)	C14—C13—N3—C12	57.8 (2)
N1—C9—C10—N2	−173.76 (18)	C14—C13—N3—C15	179.35 (18)
N2—C11—C12—N3	60.1 (2)	C16—C15—N3—C12	−168.0 (2)
N3—C13—C14—N2	−58.9 (3)	C16—C15—N3—C13	70.9 (3)
N3—C15—C16—O3	165.2 (2)		

### Hydrogen-bond geometry (Å, °)

**Table:**

*D*—H···*A*	*D*—H	H···*A*	*D*···*A*	*D*—H···*A*
O3—H3A···N3^i^	0.82	2.00	2.811 (3)	171

Symmetry codes: (i) *x*−1/2, −*y*+1/2, *z*−1/2.
